# Dysphonia risk screening protocol

**DOI:** 10.6061/clinics/2016(03)01

**Published:** 2016-03

**Authors:** Katia Nemr, Marcia Simões-Zenari, João Marcos da Trindade Duarte, Karen Elena Lobrigate, Flavia Alves Bagatini

**Affiliations:** Faculdade de Medicina da Universidade de São Paulo, Physiotherapy, Speech-Language and Hearing Sciences, and Occupational Therapy, São Paulo/SP, Brazil

**Keywords:** Voice, Voice Disorders, Speech-Language Pathology, Medical History Taking

## Abstract

**OBJECTIVE:**

To propose and test the applicability of a dysphonia risk screening protocol with score calculation in individuals with and without dysphonia.

**METHOD:**

This descriptive cross-sectional study included 365 individuals (41 children, 142 adult women, 91 adult men and 91 seniors) divided into a dysphonic group and a non-dysphonic group. The protocol consisted of 18 questions and a score was calculated using a 10-cm visual analog scale. The measured value on the visual analog scale was added to the overall score, along with other partial scores. Speech samples allowed for analysis/assessment of the overall degree of vocal deviation and initial definition of the respective groups and after six months, the separation of the groups was confirmed using an acoustic analysis.

**RESULTS:**

The mean total scores were different between the groups in all samples. Values ranged between 37.0 and 57.85 in the dysphonic group and between 12.95 and 19.28 in the non-dysphonic group, with overall means of 46.09 and 15.55, respectively. High sensitivity and specificity were demonstrated when discriminating between the groups with the following cut-off points: 22.50 (children), 29.25 (adult women), 22.75 (adult men), and 27.10 (seniors).

**CONCLUSION:**

The protocol demonstrated high sensitivity and specificity in differentiating groups of individuals with and without dysphonia in different sample groups and is thus an effective instrument for use in voice clinics.

## INTRODUCTION

An essential component of the speech-language pathologist's voice assessment is the initial investigation, which, in most cases, is designated as anamnesis. Here, a set of closed, semi-open and/or open questions is administered to aid clinicians in understanding the problem and its possible causes 1,2,3,4,5. In a voice clinic, anamnesis can be decisive in the differential diagnosis of dysphonia; in particular, it is extremely important for understanding each case and also provides the initial moment at which a bond is established between the speech therapist and the patient 6.

The data from this initial investigation have been analyzed in combination with data on other procedures, such as a laryngological evaluation, auditory-perceptual and acoustic voice analyses and a voice-related quality-of-life measure 3.

In contrast to the availability of models of voice-related quality-of-life protocols in which the scores determine the impact of dysphonia for a given individual 7,8, we did not find any protocol for an initial investigation that allows for individual assessment based on communication profile scores and that also reveals the individual's potential risk of developing a voice disorder. The currently available questionnaires are mainly restricted to the gathering of signs/symptoms and several risk factors; among these, we highlight the Voice Symptom Scale 9 and the Voice Capabilities Questionnaire 10.

In addition to assessing the signs and symptoms, other aspects related to the communicative context, including vocal self-perception using a visual analog scale (VAS) 11, can extend the initial investigation to include screening for the risk of dysphonia. Therefore, comprehensive anamnesis would offer more consistent data for both the patient and the professional; in addition, the inclusion of scores would provide objective data, which can be more readily compared with other variables. The calculation of scores also allows expanded use of this assessment tool compared with other conditions investigated in voice clinics.

A standardized and validated protocol with the calculation of a score would provide significant contributions to the study of voice 12. In the context of health assistance, such a protocol would allow classification of the patient's probability of developing a given disorder and would present a clearer contextualization of habits, signs and symptoms as well as factors that interfere with the voice. As a result, the use of a protocol can help the professional to determine the best approaches in terms of guidance, treatment plans, and/or patient follow-up in a more objective manner; it also facilitates data sharing and discussion with the patient about his or her communicative context. In the research field, a protocol allows for the comparison of results between different time points and between different care services, in addition to offering other voice assessment protocols and consistent data to guide evidence-based practice 13. Furthermore, a protocol that can be adapted to various languages and that facilitates cross-cultural comparisons can provide relevant data for both the development of activities promoting vocal well-being and for worldwide epidemiological studies. Finally, this tool can serve as a teaching instrument, thereby allowing for more systematic acquisition of knowledge in the teaching environment 14.

The present study was performed to fabricate a proposal for the study of voice that bridges the existing gap and that is also effective in differentiating symptomatic and asymptomatic individuals. Accordingly, the aim of this study was to propose and test the applicability of the Dysphonia Risk Screening Protocol (DRSP) with score calculation for individuals of different age groups with and without vocal complaints.

## METHODS

This cross-sectional study was approved by the home institution's ethics committee (CAPPEsq HCFMUSP 0560/10).

### Sample

The participants were recruited from the larger group of patients subjected to larynx examination at the Hospital das Clínicas da Faculdade de Medicina da Universidade de São Paulo (HCFMUSP), Ambulatório de Otorrinolaringologia, during the study period. In addition, the persons accompanying the patients and the professionals working in close association with the medical team were invited to participate both at the Hospital das Clínicas and at the Department of Physiotherapy, Speech-language and Hearing Science and Occupational Therapy, Faculdade de Medicina, Universidade de São Paulo.

The patients with vocal complaints were recruited concomitantly with those without complaints and efforts were made to maintain group pairings by gender and age. Upon completion of the auditory-perceptual and acoustic voice assessment described below, the presence or absence of voice disorders was analyzed and the subjects were divided into a dysphonic group (DG) and a non-dysphonic group (NDG).

Among the children and seniors, we chose to keep the participants of both genders together within their groups due to the anatomical and physiological similarities within each age group.

Regarding the inclusion criteria, all patients who agreed to participate in the study were included, regardless of their laryngological diagnosis, gender and age.

Regarding the exclusion criteria, individuals with any other impairments or diagnoses that might limit communication were excluded.

To assess the applicability and validity of the protocol, 365 subjects were enrolled in the study and were divided into four sample groups (I - children, II - adult women, III - adult men, and IV - seniors). The subjects were then further divided as follows:

I - Of 41 children, 19 children (10 girls and 9 boys) with a mean age of 7.5 years (±1.7) were allocated to the DG, and 22 children (11 boys and 11 girls) with a mean age of 8.5 years (±1.8) were allocated to the NDG.

II - Of 142 adult women, 74 women with a mean age of 41 years (±12.7) were allocated to the DG, and 68 women with a mean age of 33.9 years (±12.6) were allocated to the NDG.

III - Of 91 adult men, 41 men with a mean age of 42.8 years (±13.1) were allocated to the DG, and 50 men with a mean age of 32.2 years (±12.2) were allocated to the NDG.

IV - Of 91 senior subjects, 54 (32 women and 22 men) individuals with a mean age of 68.2 years (±6.2) were allocated to the DG, and 37 (21 women and 16 men) individuals with a mean age of 68.4 years (±7.1) were allocated to the NDG.

### Procedures

Step 1: The proposal presented here arose from the need to standardize an initial investigation protocol for use in the study of voice in teaching, research and the provision of care in FMUSP Laboratory of Voice Research. Based on adaptation of the protocol used in this laboratory, the goal was to expand the proposed protocol to provide a tool that would enable measurement via partial and total scores and that would have the ability to indicate an individual's risk of developing or suffering from dysphonia. Based on various adjustments and a pilot study with a sample of 15 patients, it was hypothesized that higher partial and/or total scores would be indicative of an increased risk of dysphonia and that lower scores would indicate a lower risk thereof. In this sense, in the first stage of the proposed general DRSP, the tool was effective in differentiating subjects with vocal complaints/disorders from individuals without voice complaints/disorders at different ages 12.

In addition to the initial questions related to personal identification, the final version of the protocol (Appendix 1) contained 18 questions that could be answered by people of any age, gender, level of education and use of voice. For the score calculation, each response score ranged from 0 to 3, with 0 representing a positive response and scores between 1 and 3 representing negative responses, ranging from least 1 to most 3 negative. The exception to this scale was a VAS, in which the value measured using a millimeter ruler was added to the overall score. Each set of questions generated a partial score and the sum of all scores yielded the total score. The partial scores were related to the sub-items comprised by the DRSP, including the following: VAS - self-assessment of voice, with a value between 0 (no disorder) and 10 (maximum disorder), for which the value calculated with the millimeter ruler was added to the final protocol value; PD - previous voice disorders; SS - signs and symptoms; VOW - use of voice outside of work; DI - diet; H - hydration; MD - medications; S - contact with smokers; SL - sleep; ILL - history of illness; FH - family history of vocal disorders; FD - family dynamics; PA - physical activities; and LS - leisure. The maximum total score was 131, which indicated an extreme risk of dysphonia.

Step 2: The individuals who agreed to participate signed an informed consent form and completed the DRSP. Speech samples were then recorded using vocal tasks predefined in the Brazilian Portuguese translation of the Consensus Auditory Perceptual Evaluation-Voice (CAPE-V) 15. The recording was performed in an acoustically treated room with noise levels below 50 dB. The desktop computer used for recording and processing was outfitted with Sound Forge Pro 10 software (Sony Inc., Japan), an audio interface (UA-101 model, Edirol by Roland, Australia), and a unidirectional condenser headset microphone (520 model, AKG, Germany). While recording the vocal tasks, the microphone was placed at a distance of 3 to 5 cm from the individual's mouth at an angle between 45° and 90°. Tests were performed to adjust the voice gain to avoid loss of peaks or very weak sounds. The participant was instructed to sit comfortably and to speak in a normal tone of voice.

Step 3: The vocal samples were classified according to the overall degree (D) of vocal deviation using the CAPE-V protocol to separate the NDG and DG; this evaluation was always performed by the same speech therapist. This speech therapist was a voice specialist with extensive experience in auditory perceptual voice assessment and showed high intra-rater reliability (intraclass correlation coefficient of 0.975).

All participants with complaints exhibited a certain degree of disorder and were confirmed to belong to the DG. No participants in the groups of women and men without complaints showed any degree of dysphonia. In the groups of children and senior subjects, those without complaints and with a mild level of change were included in the NDG. A certain degree of vocal disorder can be considered normal in childhood due to disorders in the vocal tract in terms of position, size and tissue change, which affect phonatory agility and flexibility 16,17. In seniors, slight vocal disorders, even without vocal complaints, may be related to physiological disorders, such as reduced vital respiratory capacity, maximum phonation time and muscle tone and atrophy of the intrinsic muscles, associated with a decreased muscular capacity to articulate speech sounds 18.

Step 4: To confirm the presence of disorders, the individuals in the DG underwent laryngostroboscopy, which was performed by the same team for 100% of the participants. The procedure further confirmed the homogeneity of this group. Different diagnoses were not considered in this study.

Step 5: All voice samples were analyzed using an acoustic voice analysis to confirm the results obtained in the auditory-perceptual analysis regarding the composition of the two groups. This assessment was performed by the same speech therapist six months after the auditory-perceptual analysis, using sample recordings of sustained vowel sounds, as described in Step 2. The recordings were arranged randomly by the researcher in charge, who was blinded to the previous analysis, all participant data (age, gender, diagnosis), and the groups to which the participants belonged. The analysis was performed using VoxMetria software (CTS Informática, Brazil) and included extraction of the automatically measured jitter/period perturbation quotient (PPQ) (%), shimmer/extent perturbation quotient (EPQ) (%), correlation value, irregularity, glottal-to-noise excitation (GNE) ratio and noise. Moreover, the phonatory deviation diagram (PDD) was considered with regard to the density (spread or concentrated), shape (circular, horizontal, or vertical), and quadrant (1 - normal, 2, 3, or 4 - altered) 19.

Calculation of the partial and total DRSP scores was performed without any indication of the group to which the participant belonged, and the presentation of the voices for auditory-perceptual and acoustic analyses was performed randomly for each evaluation. Similarly, the group to which the subject belonged was not identified.

The statistical analysis consisted of descriptive measures and the application of the Mann-Whitney and Chi-square tests according to the types of variables to check for differences between the DG and the NDG with respect to age, gender, being or not being a professional voice user, VAS value, sub-item partial scores and total score; the same tests were used to verify the group divisions obtained based on the acoustic analysis. A receiver operating characteristic (ROC) curve was used to define cut-off points for the total score based on the best sensitivity and specificity identified in each group. In this study, the combined use of acoustic and auditory-perceptual analyses was considered as the gold standard. A significance level of 5% was adopted. It is worth noting that all participants in the DG underwent laryngoscopy to confirm the presence of voice disorders due to different laryngological diagnoses.

Based on the laryngeal examination, DG patients showed the following diagnoses (the respective absolute numbers of women, men, seniors and children are presented): cysts (11, 3, 2, 3), chorditis (1, 3, 2, 3), Reinke's edema (11, 1,9,0), incomplete glottal closure (4, 1, 0, 3), vocal fold leukoplakia (4, 2, 8, 0), paradoxical vocal fold motion (1, 0, 0, 0), nodules (7, 0, 0, 8), papilloma (2, 4, 3, 3), bilateral vocal fold palsy (0, 0, 1, 0), unilateral vocal fold palsy (6, 5, 10, 1), polyps (6, 15, 5, 0), mucosal bridge (1, 0, 0, 0), psychogenic dysphonia (2, 0, 0, 0), laryngopharyngeal reflux (7, 1, 4, 0), sulcus bilateral (6, 1, 2, 0), unilateral sulcus (2, 1, 2, 0), vocal fold vascular lesions (1, 0, 0, 0), granuloma (0, 5, 1, 0), Parkinson's disease (0, 1, 0, 0), microweb (0, 0, 0, 1), spasmodic dysphonia (0, 0, 1, 0) and presbylarynx (0, 0, 5, 0).

## RESULTS

The gold standard for this study, which combined acoustic and auditory-perceptual voice analyses performed at six-month intervals in blinded samples, resulted in perfect differentiation between the DG and the NDG. The acoustic analysis was consistent with the findings of the auditory-perceptual analysis, given that all of the aspects considered equally separated the participants in the NDG and DG into the four sample groups: jitter (<0.001), shimmer (<0.001), the correlation value (<0.001), irregularity (<0.001), the GNE ratio (0.025 to <0.001), noise (0.021 to <0.001) and features of the PDD. Among the aspects considered in the PDD (density, shape and quadrant), the third was the feature with the highest statistical power, as 100% of the participants in the NDG were classified in quadrant 1 and 100% of the participants in the DG were placed in quadrant 2, 3, or 4.

Regarding the composition of the groups, age and gender were homogeneous in the children's and seniors' groups when comparing the DG and NDG. In the adult men's and women's groups, tendencies toward higher age were observed in the respective DGs.

Distribution into the DG and NDG according to the classification of being a professional voice user was similar in the women's, men's, and seniors' groups. This parameter did not apply in the children's group.

There were differences in VAS self-assessment (*p*<0.001) between the DG and the NDG in all age groups, with an overall mean score of 5.85 in the DG (Table 1).

Concerning the partial scores obtained according to the protocol, differences were observed between the groups for several of these scores, with greater values in the DG compared with the NDG (Tables 1 and 2).

Regarding the partial score for previous voice disorders (PD), greater means were observed in the DG in the children's, women's, and men's groups. There were no differences between the DG and the NDG in the seniors' group (Table 1).

Greater mean frequencies with which participants indicated vocal signs and symptoms (SS) were observed in the DG for all four sample groups (Table 1), with mean variations of 18.9 (children) to 35.2 (women) in the DG and 4.6 (women) to 7.0 (seniors) in the NDG.

Regarding the use of voice outside of work/school (VOW), greater scores were observed for the DG in the children's (*p*=0.002) and women's (*p*=0.050) groups. There were no differences in the men's and seniors' groups (Table 1).

In relation to dietary aspects that affect voice, there were greater scores in the DG for both men (*p*<0.001) and women (*p*<0.001). The DG and NDG had similar means in the children's and seniors' groups (Table 1).

With regard to hydration, seniors in the DG had a greater score than those in the NDG did (*p*=0.008). In contrast, in the men's, women's and children's groups, no differences were observed (Table 1).

A greater mean was recorded for the use of medicines that interfere with the voice for only the group of men in the DG (*p*=0.048). None of the children used medication that affected the voice and in the women's and seniors' groups, there were no differences between the DG and the NDG (Table 2).

Greater contact with smokers (S) in the DG was observed only for men. With regard to sleep (SL), all groups showed similar means in the DG and NDG (Table 2). Regarding history of illness (ILL), only the children's group showed similarity between the groups; in the other groups, the scores in the DG were greater (*p*<0.001) (Table 2).

The seniors' group showed a tendency toward having a family history of dysphonia (FH) and negative family dynamics (FD) more often in the DG (*p*=0.080 and *p*=0.062, respectively). Considering other ages and genders, the DG and NDG were similar with respect to FH. The same pattern was observed in the children's and men's groups for FD. However, for this sub-item, the women's DG presented negative FD more often than those in the NDG (*p*<0.001).

All groups were similar with respect to physical activity (PA). The DG showed a tendency toward lower leisure frequencies for both men (*p*=0.084) and seniors (*p*=0.052). In the children's group, both the DG and the NDG had the same leisure frequency.

The total scores revealed different means between the DG and the NDG in all sample groups. In the DG in particular, values ranged from 37.0 (children) to 57.85 (women) and in the NDG, values ranged from 12.95 (men) to 19.28 (seniors) (Table 2). The overall means were 46.09 in the DG and 15.55 in the NDG.

**Figure 1:**
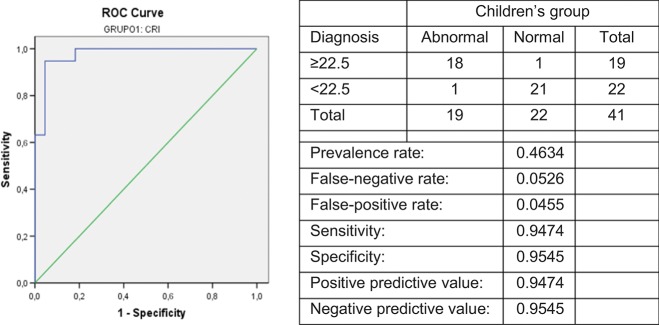
Receiver operating characteristic curve for the total Dysphonia Risk Screening Protocol scores for the children's group.

**Figure 2:**
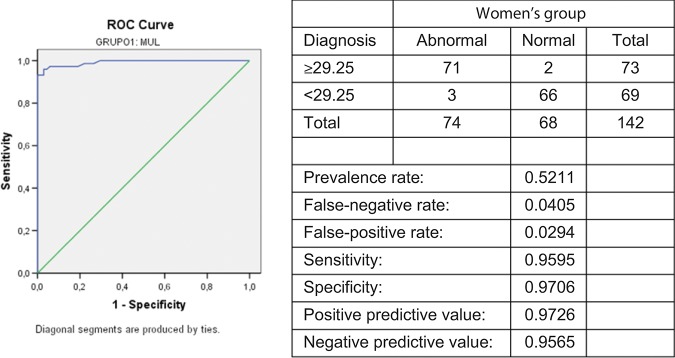
Receiver operating characteristic curve for the total Dysphonia Risk Screening Protocol scores for the women's group.

**Figure 3:**
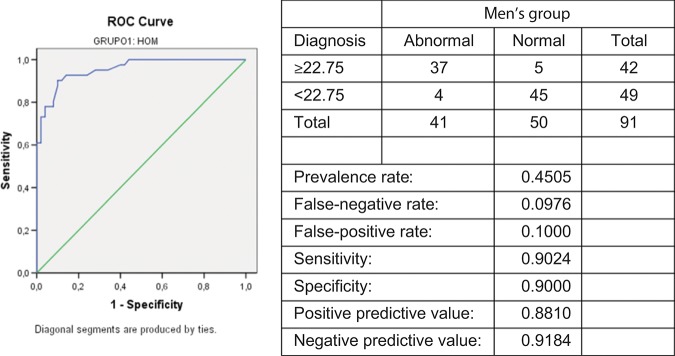
Receiver operating characteristic curve for the total Dysphonia Risk Screening Protocol scores for the men's group.

**Figure 4:**
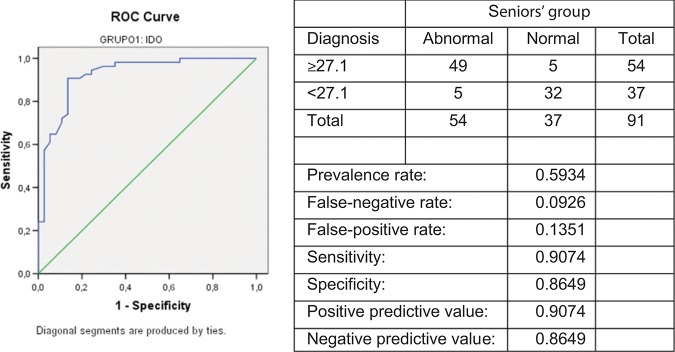
Receiver operating characteristic curve for the total Dysphonia Risk Screening Protocol scores for the seniors' group.

The ROC curves and total score cut-off points were obtained based on the calculation of the protocol's sensitivity and specificity in discriminating between the DG and the NDG (Figures 1-4) for each sample group. The following were established:

1. Children: 22.50 cut-off with a sensitivity of 0.947 and a specificity of 0.955 (Figure 1).
2. Adult women: 29.25 cut-off with a sensitivity of 0.959 and a specificity of 0.971 (Figure 2).
3. Adult men: 22.75 cut-off with a sensitivity of 0.902 and a specificity of 0.900 (Figure 3).
4. Seniors: 27.10 cut-off with a sensitivity of 0.907 and a specificity of 0.865 (Figure 4).

## DISCUSSION

The importance of testing a new tool for initial investigation that encompasses anamnesis and self-assessment within a dysphonia risk screening protocol is based on the tool's applicability from clinical, teaching and research perspectives within the study of voice. Studies have reported the use of anamnesis in voice assessment, although the tool is often not administered. Pereira et al. 4 specifically used a questionnaire consisting of demographic (age, gender) and professional (working conditions, weekly working hours and absenteeism, length of employment) data, vocal symptoms (hoarseness, throat clearing, dysphagia, difficulty singing, vocal fatigue, cough, neck ache, difficulty in voice projection, loss of vocal power) and comorbidities (nasal, gastroesophageal and auditory symptoms) but did not detail the parameters of each item considered in their research.

The standardization of tools using a scoring system allows intra- and inter-institutional comparisons to be made, along with cross-cultural research that can validate those comparisons. The voice handicap index (VHI) is a good example of intercultural adaptation, as this index was translated, adapted and validated in several languages 20,21,22. Similarly, the comparison of a certain tool across different populations or of various tools within the same sample population can contribute to the advancement of an area of knowledge 23.

In the groups of adult women and men in the current study, the age of the participants in the DG was higher than that in the NDG. It should be noted that many adult patients delay seeking medical/speech therapy care. A recent study of teachers and non-teachers with voice changes reported the predominance of both genders in the 31- to 50-year-old age group 4.

Both the DG and the NDG sample groups were homogeneous with respect to being a voice professional, which eliminated a possible bias related to voice use.

When translating and adapting the children's VHI into Hebrew, Amir et al. 3 observed that during interviews, that fathers and mothers similarly assessed the voices of children with dysphonia, although mothers were more stringent. In our study, parents could complete the DRSP for their children together; however, mothers were more likely to complete the DRSP because they accompanied their children more often. All children with vocal complaints also presented with auditory perceptual deviation, reflecting the adequate perception of the parents.

Regarding seniors, even though those without voice changes and those with mild changes were included in the DG, all seniors without changes were further tested and compared with seniors with changes, regardless of the degree of the change (mild, moderate, or severe), there was also differentiation between groups. It should be noted that in seniors, slight disorders might not simply be due to presbyphonia; such deviations could also be due to the onset of neurological disease. These possible changes should be examined and the DRSP is an instrument that is able to detect such differences. In one patient referred for speech therapy, initial investigation and vocal assessment data contributed to the diagnosis of myasthenia gravis associated with presbyphonia, which was the reason for referral 24.

Regarding the VAS-based voice self-assessment, all groups exhibited perceptions of their voices that were compatible with the study hypothesis, as the DG had greater means than the NDG did. One of the innovations proposed in the present study is the inclusion of the VAS in the questionnaire and the addition of the VAS score to the overall score.

Regarding a history of previous voice changes, Yamauchi et al. 18 used high-speed laryngoscopy to compare healthy vocal fold vibrations with the vibrations of vocal cords with atrophy, revealing higher open quotients, greater lateral phase differences and a greater integral glottal width in the latter group. The authors noted that the incidence rates of voice disorders in subjects with vocal fold atrophy are increasing rapidly due to the aging of populations worldwide. These observations do not necessarily relate to the use of the voice throughout life and may explain the fact that the senior DG did not present a history of previous voice changes more frequently than the NDG did in the current study.

Regarding the frequency of vocal signs/symptoms, partial scores were higher for the four DGs, consistent with previous studies that have also considered frequency. Other Brazilian studies have similarly reported significant differences between groups with and without dysphonia 25,26.

In voice clinics, vocal signs/symptoms are included in the first questionnaires given to the patient. Moreover, the retrieval of such information is very frequent during screening and voice care programs and is achieved in many ways. In particular, the literature describes the Voice Symptom Scale 9 as a rigorous and psychometrically robust tool for vocal self-assessment and provides information on the functional effects, emotional impact and physical effects that a voice problem may have on an individual's life 26,27. The Voice Capabilities Questionnaire 10 is also used to measure vocal symptoms, but without implying that the respondent necessarily has a voice disorder. The proposed DRSP falls within this same line of investigation and offers the potential to survey broader aspects of the voice. This assessment may be used in individuals with or without dysphonia, with varying gradations as to the potential risks of developing vocal disorders in the latter case. The complex nature of voice assessment and the need for appropriate tools, especially for functionally healthy voice professionals, merit attention 28. Another aspect that should be highlighted is the possibility of spontaneous inclusion of other symptoms in the DRSP in addition to those listed.

Regarding the use of the voice outside of work/school (VOW) in the present study, the higher scores in the DG for the children's and women's groups were related to the greater predisposition to the development of vocal nodules in these populations, especially if associated with laryngeal tension and vocal abuse 29.

The greater scores in the DG observed for the men's and women's groups regarding dietary aspects related to the voice draw attention to current sociocultural issues, wherein a productive adult life, especially in large cities, has fostered the development of poor eating habits that affect health and have possible vocal consequences. This finding corroborates the high prevalence of voice disorders associated with laryngopharyngeal reflux 30.

In the present study, hydration was present with greater frequency in the NDG, though only for the seniors' group. A recent literature review indicated that although several studies in the voice field suggest a relationship between hydration and vocal function, there is a need for greater understanding of this aspect to guide best practices in health maintenance and the prevention of voice disorders 31.

The difference found for the men's group in the current study should be further investigated because the protocol provides detailed information regarding the type and dosage of drugs used, which is information that was not considered here. Therefore, one should consider not only the medications prescribed to treat the clinical conditions that affect the voice but also those that can increase the potential risk of dysphonia 32,33.

The association detected between the presence of dysphonia and greater contact with smokers in the men's group in this study requires further elucidation. Because the DRSP is a general protocol designed for use by people of any age, gender, or professional voice use status, it does not contain questions regarding smoking itself; instead, these questions appear in the supplementary DRSP, which is applicable only to adults and seniors.

Regarding history of illness, the greater scores in all DGs apart from the children's DG may have been due to comorbidities associated with dysphonia 30,.

The relationship between negative family dynamics and dysphonia in the women's group corroborates the aspects mentioned in relation to intense voice use and the greater predisposition of this population to the development of vocal nodules 29. This association also indicates the negative emotional impact of family relationships in this group.

During analysis of the results, when the scores for questions that did not differentiate between the DG and the NDG across the four sample groups were removed following calculation, the means continued to discriminate the groups to similar degrees. Because these questions involved qualitative aspects relevant to the communicative context and because benefits were not observed following their removal from the score, these questions were retained.

As a continuation of the present study, proposals to supplement the DRSP with specific protocols for children, for spoken and singing voice professionals and for adults and seniors are under development. It is believed that the application of both general and specific DRSPs will allow the gathering of thorough knowledge about dysphonia risk for particular groups.

Based on the greater sensitivity and specificity values of the DRSP in the four sample groups, it was possible to establish cut-off points in the total scores; this further reinforces the importance of the presented protocol for the study of voice, not only in the clinic but also in health promotion programs and in the field of epidemiology. The protocol used will additionally enable the study of the multiplicity of factors involved in the etiology of dysphonia.

The DRSP is therefore an instrument complementary to auditory-perceptual, acoustic and physiological voice evaluations. However, isolated analysis of this instrument should be considered with caution.

It should be noted that patients under treatment at the Laboratory of Voice/Speech Research at USP, with controls employed before, during and after speech therapy, have exhibited shifts in the therapeutic process based on DRSP scores. For example, one patient with progressive worsening of Parkinson's disease presented a gradual increase in the final DRSP score; in contrast, patients with initially high scores have presented lower final scores. These results encourage further studies to confirm this trend, with the possibility of risk classification.

By employing score calculations and applying them to different age groups, the DRSP was found to exhibit high sensitivity and specificity in differentiating groups of individuals with and without dysphonia.

The DRSP thus proved to be an effective instrument for use in voice clinics.

## AUTHOR CONTRIBUTIONS

Nemr K was responsible for the research and experimental design and contributed to the data analysis and manuscript preparation. Simões-Zenari M supervised the data collection and contributed to the data analysis and manuscript preparation. Duarte JM, Lobrigate KE and Bagatini FA participated in the data collection and contributed to the data analysis.

## APPENDIX 1

## Figures and Tables

**Figure:**
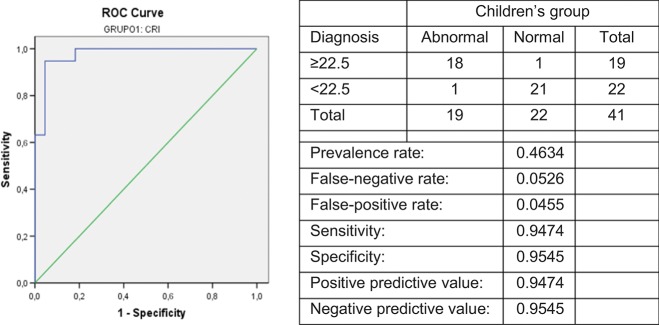


**Table 1:** Data distribution regarding partial Dysphonia Risk Screening Protocol scores (respective scores for visual analog scale, previous voice disorders, signs and symptoms, use of voice outside of work, diet and hydration shown) for the four sample groups.

Analyzed aspect	Groups		Mean	Median	Standard deviation	*p* value
VAS	Children	NDG	0.6	0.0	1.2	<0.001*
				DG	5.9		6.0	3.0
		Women	NDG	0.8	0.0	1.1	<0.001*
				DG	6.3	6.1		2.3
		Men	NDG	1.0	0.3	1.3	<0.001*
				DG	5.0	5.0		2.6
		Seniors	NDG	1.5	1.8	1.9	<0.001*
				DG	6.2	6.0		2.7
PD	Children	NDG	0.1	0.0	0.4	<0.001*
				DG	1.0		1.0	0.9
		Women	NDG	0.0	0.0	0.2	<0.001*
				DG	0.7	1.0		0.6
		Men	NDG	0.2	0.0	0.5	0.010*
				DG	0.4	0.0		0.7
		Seniors	NDG	0.3	0.0	0.5	0.092
				DG	0.5	0.0		0.6
SS	Children	NDG	4.9	4.5	4.5	<0.001*
				DG	18.9		18.0	9.8
		Women	NDG	4.6	4.0	4.1	<0.001*
				DG	35.2	33.0		16.1
		Men	NDG	5.0	3.0	4.9	<0.001*
				DG	24.2	21.0		14.5
		Seniors	NDG	7.0	4.0	8.1	<0.001*
				DG	29.4	26.0		13.7
VOW	Children	NDG	4.9	4.5	4.5	0.002*
				DG	18.9		18.0	9.8
		Women	NDG	4.6	4.0	4.1	0.050*
				DG	35.2	33.0		16.1
		Men	NDG	5.0	3.0	4.9	0.189
				DG	24.2	21.0		14.5
		Seniors	NDG	7.0	4.0	8.1	0.159
				DG	29.4	26.0		13.7
DI	Children	NDG	1.3	1.0	1.0	0.122
				DG	2.0		2.0	1.5
		Women	NDG	1.7	2.0	1.3	<0.001*
				DG	2.9	3.0		1.5
		Men	NDG	1.1	1.0	0.9	<0.001*
				DG	2.2	2.0		1.1
		Seniors	NDG	2.3	2.0	1.4	0.954
				DG	2.3	2.0		1.6
H	Children	NDG	1.5	2.0	0.9	0.504
				DG	1.2		2.0	1.1
		Women	NDG	1.4	2.0	0.9	0.357
				DG	1.8	2.0		2.7
		Men	NDG	0.7	0.0	1.0	0.251
				DG	1.0	1.0		1.1
		Seniors	NDG	1.5	2.0	1.4	0.008*
				DG	2.3	3.0		1.5

**<?ENTCHAR ast?>:** Statistically significant; Mann-Whitney test

**Table 2:** Data distribution regarding partial Dysphonia Risk Screening Protocol scores (respective scores for medications, contact with smokers, sleep and history of illness shown) and total scores in the four sample groups.

Analyzed aspect	Groups		Mean	Median	Standard deviation	*p* value
MD	Children	NDG	0.0	0.0	0.0	1.000
				DG	0.0		0.0	0.0
		Women	NDG	0.4	0.0	0.4	0.557
				DG	0.4	0.0		0.5
		Men	NDG	0.0	0.0	0.2	0.048*
				DG	0.2	0.0		0.4
		Seniors	NDG	0.6	1.0	0.5	0.970
				DG	0.6	1.0		0.5
S	Children	NDG	0.4	0.0	0.9	0.170
				DG	1.1		0.0	1.4
		Women	NDG	0.5	0.0	0.8	0.091
				DG	0.8	0.0		1.1
		Men	NDG	0.5	0.0	0.9	0.032*
				DG	0.8	1.0		1.0
		Seniors	NDG	0.5	0.0	0.8	0.530
				DG	0.6	0.0		0.9
SL	Children	NDG	0.3	0.0	0.6	0.210
				DG	0.5		0.0	0.6
		Women	NDG	2.3	2.0	1.8	0.513
				DG	2.5	2.0		1.8
		Men	NDG	0.9	1.0	1.0	0.101
				DG	1.2	1.0		0.9
		Seniors	NDG	1.2	1.0	0.9	0.826
				DG	1.1	1.0		1.0
ILL	Children	NDG	1.0	1.0	0.9	0.900
				DG	1.0		1.0	0.9
		Women	NDG	0.8	0.0	1.0	<0.001*
				DG	2.6	3.0		1.5
		Men	NDG	1.0	1.0	0.9	<0.001*
				DG	2.0	2.0		1.2
		Seniors	NDG	1.4	1.0	1.3	<0.001*
				DG	2.7	3.0		1.8
Total score	Children	NDG	13.9	13.0	6.4	<0.001*
				DG	37.0		39.0	11.1
		Women	NDG	16.0	14.8	6.7	<0.001*
				DG	57.8	57.2		18.9
		Men	NDG	12.9	11.0	6.7	<0.001*
				DG	40.3	39.0		16.5
		Seniors	NDG	19.2	16.0	12.1	<0.001*
				DG	49.2	48.2		17.1

**<?ENTCHAR ast?>:** Statistically significant; Mann-Whitney test

## References

[1] Goulart BNG, Chiari BM (2007). Avalia&ccedil;&atilde;o cl&iacute;nica fonoaudiol&oacute;gica, integralidade e humaniza&ccedil;&atilde;o: perspectivas gerais e contribui&ccedil;&otilde;es para reflex&atilde;o. Rev Soc Bras Fonoaudiol.

[2] Oliveira IB Ferreira L P, Avalia&ccedil;&atilde;o fonoaudiol&oacute;gica da voz: reflex&otilde;es sobre condutas, com enfoques &agrave; voz profissional Tratado de Fonoaudiologia 2004 São Paulo Roca 11 23

[3] Amir O, Wolf M, Mick L, Levi O, Primov-Fever A (2015). Parents&apos; evaluations of their children&apos;s dysphonia: the mamas and the papas. J Voice.

[4] Pereira ER, Tavares EL Martins RH (2015). Voice disorders in teachers: clinical, videolaryngoscopical, and vocal aspects. J Voice.

[5] Simberg S, Udd H, Santtila P (2015). Gender differences in the prevalence of vocal symptoms in smokers. J Voice.

[6] Behlau M Rodrigues S Azevedo R Gonçalves MI Pontes P Lopes Filho O, Avaliação e terapia de voz 2005 São Paulo Tecmedd editorTratado de Fonoaudiologia

[7] Jacobson HB, Johnson A, Grywalski C, Silbergleit AK, Jacobson GP, Benninger M (1997). The Voice Handicap Index (VHI): development and validation. Am J Speech Lang Pathol.

[8] Hogikyan ND, Sethuraman G (1999). Validation of an instrument to measure voice-related quality of life (V-RQOL). J Voice.

[9] Deary IJ, Wilson JA, Carding PN, MacKenzie K (2003). VoiSS: a patient-derived Voice Symptom Scale. J Psychosom Res.

[10] Buckley KL, O'Halloran PD, Oates JM (2015). Occupational vocal health of elite sports coaches: an exploratory pilot study of football coaches. J Voice.

[11] Gama ACC, Camargo Z, Santos MAR, Rusilo LC (2015). Discriminant capacity of acoustic, perceptual, and vocal self: the effects of vocal demands. J Voice.

[12] Simões-Zenari M, Bonfim ACC, Nemr K (2011). Proposta de protocolo de anamnese fonoaudiol&oacute;gica para a cl&iacute;nica de voz. Rev Soc Bras Fonoaudiol.

[13] American Speech-Language-Hearing Association (2005). Evidence-Based Practice in Communication Disorders [Internet].

[14] Medina V, Simões-Zenari M, Nemr NK (2015). An&aacute;lise vocal ac&uacute;stica: efeito do treinamento auditivo-visual para graduandos de Fonoaudiologia. Audiol Commun Res.

[15] Behlau M (2004). Consensus Auditory-Perceptual Evaluation of Voice (CAPE-V), ASHA 2003. Refletindo sobre o novo/New reflexions. Rev Soc Bras Fonoaudiol.

[16] Hersan R, Behlau M (2000). Behavioral management of pediatric dysphonia. Otolaryngol Clin North Am.

[17] Simões-Zenari M, Nemr K, Behlau M (2012). Voice disorders in children and its relationship with auditory, acoustic and vocal behavior parameters. Int J Pediatr Otorhinolaryngol.

[18] Yamauchi A, Yokonishi H, Imagawa H, Sakakibara KI, Nito T, Tayama N (2015). Quantitative analysis of digital videokymography: a preliminary study on age- and gender-related difference of vocal fold vibration in normal speakers. J Voice.

[19] Pifaia LR, Madazio G, Behlau M (2013). Phonatory Deviation Diagram pre and post vocal rehabilitation. CoDAS.

[20] Nawka T, Wiesmann U, Gonnermann U (2003). Validation of the German version of the Voice Handicap Index. HNO.

[21] Behlau M, Santos LMA, Oliveira G (2011). Cross-cultural adaptation and validation of the Voice Handicap Index into Brazilian Portuguese. J Voice.

[22] Karlsen T, Grieg ARH, Heimdal JH, Aarstad HJ (2012). Cross-cultural adaption and translation of the Voice Handicap Index into Norwegian. Folia Phoniatr Logop.

[23] Nemr K, Simões-Zenari M, Cordeiro GF, Tsuji DH, Ogawa AI, Ubrig M (2012). GRBAS and CAPE-V scales: high reliability and consensus when applied at different times. J Voice.

[24] Nemr NK, Simões-Zenari M, Ferreira TS, Fernandes HR, Mansur LL (2013). Dysphonia as the primary complaint in a case of myasthenia gravis: diagnosis and speech therapy. Codas.

[25] Ghirardi ACAM, Ferreira LP, Giannini SPP, Latorre MRDO (2013). Screening Index for Voice Disorder (SIVD): development and validation. J Voice.

[26] Moreti F, Zambon F, Oliveira G, Behlau M (2014). Cross-Cultural adaptation, validation, and cutoff values of the Brazilian version of the Voice Symptom Scale-VoiSS. J Voice.

[27] Branski RC, Cukier-Blaj S, Pusic A, Cano SJ, Klassen A, Mener D (2010). Measuring quality of life in dysphonic patients: a systematic review of content development in patient-reported outcomes measures. J Voice.

[28] Ilomäki I, Leppänen K, Kleemola L, Tyrmi J, Laukkanen AM, Vilkman E (2009). Relationships between self-evaluations of voice and working conditions, background factors, and phoniatric findings in female teachers. Logoped Phoniatr Vocol.

[29] Smillie I, McManus K, Cohen W, Lawson E, Wynne DM (2014). The paediatric voice clinic. Arch Dis Child.

[30] Spantideas N, Drosou E, Karatsis A, Assimakopoulos D (2015). Voice disorders in the general greek population and in patients with laryngopharyngeal reflux. prevalence and risk factors. J Voice.

[31] Hartley NA, Thibeault SL (2014). Systemic hydration: relating science to clinical practice in vocal health. J Voice.

[32] Ruiz R, Achlatis S, Sridharan S, Wang B, Fang Y, Branski RC (2014). The effect of antireflux therapy on phonomicrosurgical outcomes: a preliminary retrospective study. J Voice.

[33] Hackenberg S, Hacki T, Hagen R, Kleinsasser NH (2010). Voice disorders in asthma. Laryngorhinootologie.

[34] Nemr K, Simões-Zenari M, Marques SF, Cortez JP, Silva AL (2010). Disfonia psicog&ecirc;nica associada a outras doen&ccedil;as: desafio para o tratamento fonoaudiol&oacute;gico. Pró-Fono R. Atual.

[35] Schalling E, Gustafsson J, Ternstrom S, Wilen FB, Sodersten M (2013). Effects of tactile biofeedback by a portable voice accumulator on voice sound level in speakers with Parkinson's disease. J Voice.

